# An Unusual Symptom

**Published:** 1890-03

**Authors:** Edward T. Balch

**Affiliations:** South Bend, Washington


### AN UNUSUAL SYMPTOM.

EDWARD T. BALCH, M. D., SOUTH BEND, WASHINGTON.

"Doctor, can't you relieve my daughter from this sweating? She is obliged to keep her face covered to prevent sweating. I have to wipe her face continually if uncovered, and to cover it up she smothers so. Her hands and feet sweat just the same if the clothes get off. It smells so disagreeable, just like fish-pickle. Can't you do something to stop it?" Such was the urgent request made by the mother of N. W., æt. nineteen, blonde, primipara, who had just passed through a tedious and severe accouchement, and who, notwithstanding my best efforts and most careful nursing, had thus far failed to rally from her prostration; her recuperative powers seemed to lie dormant. Secretory functions subnormal. Wasting in excess of repair. Peevish, obstinate, "didn't want to be well any more, didn't care." There seemed to be an entire lack of vitality, or power to arouse "and make an effort" in her own behalf. Indeed, the case, like Mrs. Dombey, was fast assuming a serious aspect. Friends were getting anxious for a change of result or treatment, even the *sage femme* thought that more and stronger medicine, just like Dr. —— gave, was necessary. I had burned the midnight oil, determined, if possible, to find a remedy that would benefit and save the case, and rescue my fast-waning reputation from the stigma of not giving stronger medicine; had carefully and diligently watched for some ray of light, some guiding symptom that would assist me to select the simillimum, but as yet in vain, when lo! the mother detects for me a symptom that in my blind zeal I had overlooked. A symptom that had never occurred before to me in my professional life. *Sweat on uncovered parts.* I knew enough not to spoil a case by giving an unsuitable remedy, so prescribed Sac-lac. in full doses, and quieted the mother's alarm by intimating that the sweat, although an unpleasant symptom, was not necessarily a serious one and that I had left "something to stop it," at once hurried home and began a diligent search. Allen, Hoyne, Winterburn, and Hering gave no light. When, eureka! in Lippe, sweat on uncovered parts, Thuja. Water to the wounded soldier was not more welcome than was this light to me. Hastened back to my patient, and at once gave Thuja[30], a dose dry on tongue, continued Sac-lac. as before. This was at three P. M.; at nine P. M. she could sleep without covering face, next day became more cheerful, and passed to a rapid convalescence—she is now in vigorous health.

Was sycosis latent in the case, and did it prevent recovery? Would any other remedy have accomplished the same result?

